# Inflammatory responses to acute pneumovirus infection in neonatal mice

**DOI:** 10.1186/1743-422X-7-320

**Published:** 2010-11-15

**Authors:** Cynthia A Bonville, Catherine Ptaschinski, Caroline M Percopo, Helene F Rosenberg, Joseph B Domachowske

**Affiliations:** 1 Department of Pediatrics, SUNY Upstate Medical University, Syracuse, NY, USA; 2 School of Biomedical Sciences, University of Newcastle, Newcastle, NSW, 2300, Australia; 3 Laboratory of Allergic Diseases, National Institute of Allergy and Infectious Diseases, National Institutes of Health, Bethesda, MD, USA; 4 Department of Pediatrics, SUNY Upstate Medical University, Syracuse, NY, USA

## Abstract

**Background:**

The innate immune responses of neonates differ dramatically from those of adults. Here we examine the acute inflammatory responses of neonatal and weanling mice infected with pneumonia virus of mice (PVM), a rodent pathogen (family *Paramyxoviridae*, genus Pneumovirus) that replicates the sequelae of severe respiratory syncytial virus infection.

**Results:**

We demonstrate that virus replication proceeds indistinguishably in all age groups (inoculated at 1, 2, 3 and 4 weeks of age), although inflammatory responses vary in extent and character. Some of the biochemical mediators detected varied minimally with age at inoculation. Most of the mediators evaluated demonstrated elevated expression over baseline correlating directly with age at the time of virus inoculation. Among the latter group are CCL2, CCL3, and IFN-γ, all cytokines previously associated with PVM-induced inflammatory pathology in mature mice. Likewise, we detect neutrophil recruitment to lung tissue in all age groups, but recruitment is most pronounced among the older (3 - 4 week old) mice. Interestingly, all mice exhibit failure to thrive, lagging in expected weight gain for given age, including the youngest mice that present little overt evidence of inflammation.

**Conclusions:**

Our findings among the youngest mice may explain in part the phenomenon of atypical or minimally symptomatic respiratory infections in human neonates, which may be explored further with this infection model.

## Background

Nearly all aspects of immune function are distinct in newborn infants when compared to adults of a given species. Innate immune responses among mammalian neonates are typically skewed toward the production of Th2-type cytokines; the relatively limited capacity for a Th1 response (TNF, IL-12, IFNγ) has been interpreted as functionally adaptive, serving to protect the developing fetus and neonate against hyperinflammation and/or destructive responses to maternal tissues (reviewed in [1-4]). As such, neonates are particularly vulnerable to infectious diseases, as they are without adequate defense against pathogenic bacteria and viruses, and, if infected, they are potentially predisposed to allergic sequelae [5,6].

As part of our ongoing interest in innate immune responses to respiratory viral pathogens, we have characterized the pneumonia virus of mice (PVM) infection model, which replicates the pathogenesis of severe human respiratory syncytial virus (RSV) infection responses in inbred strains of mice [7]. PVM replicates in bronchial epithelial cells, inducing a profile of early pro-inflammatory mediators, including CCL2, CCL3, and IFNγ, that are associated with respiratory dysfunction and promote recruitment of inflammatory cells to lung tissue [8-10]. To date, we have characterized the biochemical and cellular responses of adult mice (8-12 week old) during infection. In this work, we examine the innate immune responses to PVM infection in newborn (1 and 2 week old) and weanling (3 and 4 week old) mice, as these hosts may more appropriately parallel the human population primarily susceptible to severe RSV infection [11]. We report our findings on virus replication as well as biochemical and cellular inflammatory responses to acute PVM infection in this critical target population, which reveal an intriguing parallel between neonatal PVM infection and atypical RSV infection in newborn humans.

## Results

### Virus recovery from lung tissue of PVM-infected neonatal and weanling mice

All mice received a minimal volume inoculum (10 μL) containing 200 pfu PVM. We found that age at inoculation had no impact on virus recovery [Table 1]. Virus recovery increased appropriately over time (day 4 vs. day 7 after inoculation), as one would anticipate for an actively replicating pathogen, but no significant differences between groups (age at time of inoculation) were detected. Virus was undetectable by day 14 among survivors from each group evaluated (data not shown).

**Table 1:** Virus recovery (PVM_SH_/10^6 ^GAPDH) from lung tissue.

	Virus recovery ( copies PVM_SH _/ 10^6 ^GAPDH)			
Age at inoculation	4 days after inoculation	n	7 days after inoculation	n
**1 week**	41 ± 8.8	6	1900 ± 203	12
**2 weeks**	43 ± 6.7	12	1570 ± 147	21
**3 weeks**	46 ± 5.9	8	1830 ±218	6
**4 weeks**	48 ± 9.4	7	1800 ± 132	10

### Differential expression of pro-inflammatory mediators

**Figure 1:**
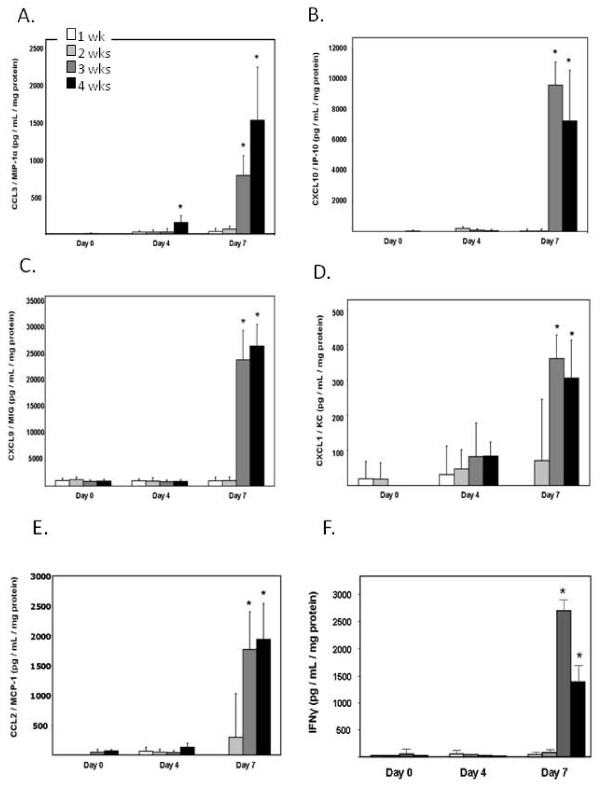
**Proinflammatory mediators expressed in lung tissue in response to PVM infection**. Detection of immunoreactive (A) CCL3 (B) CXCL10 (C) CXCL9 (D) CXCL1 (E) CCL2 and (F) IFNγ in response to PVM infection in mice at 1 week (white bars), 2 weeks (light gray bars), 3 weeks (dark gray bars) or 4 weeks old (black bars) at time of virus inoculation. Detection of immunoreactive protein is shown at days 0, 4, and 7 after inoculation for all mice. Statistical significance, *p < 0.05 vs. mediator levels of mice from younger age groups (inoculated at 1 or 2 weeks old), evaluated at day 7; n = 4 - 6 mice per group.

Differential expression (ie...expression in lung tissue of PVM-infected mice vs. expression in lung tissue of control mice) of transcripts encoding pro-inflammatory mediators was examined at day 7 after inoculation. These differential responses can be divided into two distinct groups [Table 2]: Group I includes differential responses that vary minimally (or not at all) with age at inoculation. These differential responses (including transcripts encoding CCL1, CCL6, CXCL11, and CXCL12) not only vary minimally with age at inoculation, the differential responses themselves are minimal, demonstrating at most 2-fold induction in response to virus infection. In contrast, Group II includes differential responses that increase in association with increasing age at inoculation. A good example of a Group II differential response is interferon-gamma (IFNγ), in which we observe 1.6-fold differential expression among the mice inoculated at 1 week of age, 1.9-fold at 2 weeks of age, 18.4-fold at 3 weeks of age, and 26-fold differential expression among the mice inoculated at 4 weeks of age. Others included in Group II include CCL2, CCL3, CXCL1, CXCL9 and CXCL10, which are all chemokines implicated in inflammatory pathology in response to PVM infection. These age-dependent differential responses established by PCR array were confirmed by detection of immunoreactive protein in lung tissue [Figure 1].

**Table 2:** Differential inflammatory responses.

Age at Inoculation	1 wk	2 wks	3 wks	4 wks
**Group I: **Differential responses vary minimally with age at inoculation				
CCL1 (TCA-3)	1.2	1.4	2.1	1.6
CCL6 (C10)	1.5	1.7	0.9	2.0
CXCL11 (I-Tac)	1.1	0.6	1.0	0.9
CXCL12	1.0	1.5	0.9	0.7
**Group II: **Differential responses increase with age at inoculation				
CCL2 (MCP-1)^a^	0.6	0.1	1.5	1.9
CCL3 (MIP-1α)^a^	1.9	2.2	9.8	9.2
CCL4 (MIP-1β)	1.3	1.5	9.8	11.3
CCL5 (RANTES)	1.8	2.9	3.5	4.9
CCL7 (MCP-3)	1.1	1.2	5.8	7.5
CCL8 (MCP-2)	2.2	4.4	1.4	12.1
CCL9 (MIP-1γ)	1.3	1.5	1.4	4.9
CCL11 (eotaxin)	1.9	1.1	2.3	4.3
CCL12 (MCP-5)	1.0	1.9	0.9	3.7
CCL17 (TARC)	1.2	0.9	1.0	14.9
CCL19 (MIP-3β)	2.1	0.9	1.3	29.9
CCL24 (eotaxin 2)	0.8	1.7	0.9	2.1
CXCL-1 (KC)^a^	1.4	1.4	0.7	6.5
CXCL9 (MIG)^a^	2.5	1.6	78.8	84.4
CXCL10 (IP-10)^a^	2.2	2.1	36.8	45.3
CXCL13	1.3	1.8	1.3	7.0
TNF	1.7	1.4	4.6	2.2
IFNγ^a^	1.6	1.9	18.4	26.0

Expression of proinflammatory mediators detected by PCR array analysis of RNA from lung tissue of infected mice vs. RNA from lung tissue from age-matched uninfected controls; t = day 7 after inoculation. Ages of mice at time of inoculation are as indicated; ^a^corresponding immunoreactive protein shown in Figure 1.

### Leukocyte recruitment and histopathology in PVM-infected neonatal and weanling mice

**Figure 2:**
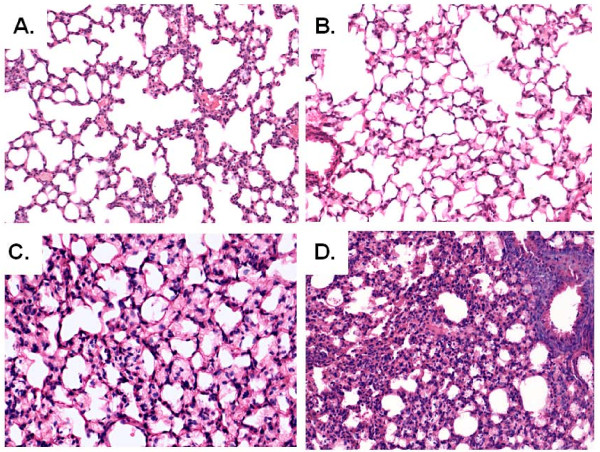
**Histopathologic analysis**. Hematoxylin and eosin-(H&E) stained lung tissue from mice inoculated with PVM at (A) 1 week (B) 2 weeks (C) 3 weeks or (D) 4 weeks of age. Lung tissue sample was taken at day 7 after inoculation; original magnification, 10×.

Leukocyte recruitment in response to PVM infection was evaluated as fold-increase over diluent-inoculated control [Table 3]. We detected prominent recruitment of neutrophils (CD11c^lo ^Gr1^+^) and CD8^+^T cells (CD3^+^CD4^-^CD8^+^) in mice inoculated at four weeks of age. As shown in Figure 2, lung tissue of 1 - 2 week old mice inoculated with PVM display little to no inflammatory pathology (day 7). In contrast, mice inoculated at 3 to 4 weeks of age display significant alveolitis at the day 7 time point, consistent with the biochemical [Table 2] and cellular [Table 3] inflammatory profiles previously described.

**Table 3:** Leukocyte recruitment in response to PVM infection.

		Age at inoculation			
Cell type - Ag profile		1 week	2 weeks	3 weeks	4 weeks
PMN	CD11c^lo ^Gr1^hi^	1.5	1.9	1.8	3.2
MØ	CD11c^+ ^CD11b^-^	1.0	1.8	1.8	1.8
mDC	CD11c^+ ^CD11b^+^	1.0	1.4	1.6	1.7
pDC	CD11c^lo ^Gr1^+ ^B220^+^	1.1	1.9	2.1	1.7
CD4^+ ^T cell	CD3^+ ^CD4^+ ^CD8^-^	1.0	1.2	1.4	1.4
CD8^+ ^T cell	CD3^+ ^CD4^- ^CD8^+^	1.0	1.1	2.3	2.4
B cells	CD3^+ ^CD19^+^	0.9	1.9	1.4	1.5

Data shown represent fold-increase over number of cells detected in age-matched mice inoculated with diluent control; n = 4 mice per condition, t = day 7 after inoculation. PMN, neutrophils; MØ, macrophages; mDC, myeloid dendritic cells; pDC, plasmacytoid dendritic cells.

### Weight gain and virus recovery in PVM-infected neonatal and weanling mice

**Figure 3:**
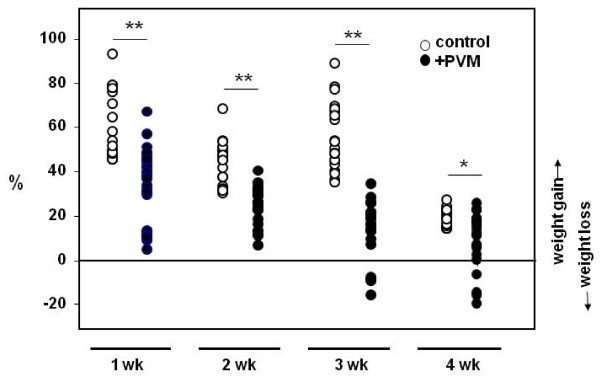
**Acute PVM infection results diminished growth**. Mice were inoculated with 10 μL/200 pfu PVM J3666 (filled symbols) or phosphate buffered-saline control (open symbols) at 1, 2, 3, or 4 weeks of age as shown. Weight was evaluated at day 0 and at day 7; percent (%) change was measured as [(weight day 7 - weight day 0) × 100/weight day 0.]. Net weight loss was observed in some PVM-infected weanling mice (7 of 44); statistical significance, *p < 0.05, **p < 0.005; n = 19 - 31 mice per group.

Normal uninfected neonatal and weanling mice undergo significant growth over the course of a single week. Mice infected with PVM at 1, 2 or 3 weeks of age exhibit substantially diminished weight gain over the ensuing one week period. For example, one week old mice infected with PVM have gained an average of 32% body weight by 7 days post-inoculation, at the peak of virus recovery; meanwhile, their uninfected counterparts have increased their body weight by 60% (p < 0.05; [Figure 3]) By 4 weeks of age, growth rate of uninfected mice has diminished; accordingly, PVM infection in these mice did not have as substantial an impact on body weight. By day 10 after inoculation, weight gain resumed in all age groups (data not shown). However, the crucial point is that all PVM-infected mice exhibit failure to thrive, even the youngest mice that experience minimal biochemical and cellular inflammation.

## Discussion

In this work, we show that acute inflammatory responses to PVM infection vary substantially with age at inoculation, which are significantly more robust among the older mice in our study; the responses of the mice inoculated at 4 weeks of age are consistent with those described previously in our earlier studies of adult (6 - 8 week old) mice [7-10]. Although several studies have documented Th2-skewing and secondary responses to virus pathogens in newborn and neonatal mice [12-14], there are few systematic evaluations of primary inflammatory responses to these virus pathogens during normal neonatal development. As such, it is interesting to compare our findings with those from a recent study of bovine RSV pathogenesis, in which the authors compared the responses of experimentally-inoculated neonatal (1 day old) and 6 week old immunologically-naïve calves to acute infection [15]. The two groups display similar peak virus recoveries, but, likewise similar to our results, the neonatal calves experienced limited TNF-alpha expression and neutrophil recruitment in response to acute virus infection.

Our finding that PVM-associated inflammatory responses in the youngest mice are dramatically different from those of older juvenile mice provides substantial insight into a long-standing clinical observations regarding neonatal hRSV infection in humans. Specifically, infants who develop hRSV bronchiolitis beyond the neonatal period develop the telltale symptom complex of nasal congestion, tachypnea, and diffuse expiratory wheezing, much of which is thought to be caused by virus-induced inflammatory responses. In contrast, human newborns infected with RSV often do not develop a wheezing illness, but instead present with nonspecific signs of illness such as temperature instability, poor feeding, periodic breathing, or apnea. The atypical nature of RSV infection in these young newborns was first described by Hall and colleagues [16]. In this cohort, nearly half of the RSV-infected newborns had lethargy, a third presented with poor feeding, and 15% had apnea episodes; cough, fever and wheezing were absent. Among the interpretations provided, Hall and colleagues suggested that the atypical symptom complex may result from the inability to mount a robust inflammatory response. These observations were mirrored by those of Wilson and colleagues [17] who described a similar symptom complex in a neonatal intensive care unit outbreak of RSV infection, and our recent study of asymptomatic respiratory virus infection among neonatal intensive care unit patients [*manuscript in review*].

Given the blunted inflammatory responses observed in neonates, it is important to consider what other factors might be promoting respiratory or even systemic illness in this uniquely susceptible target population. Among humans, one might consider the role of maternal antibodies against the RSV pathogen, which have been explored as promoting protection and in vaccination strategies [18-22]. Interestingly, as the mice used in this study were born to immunologically naïve mothers, the differences in inflammatory pathology observed cannot be attributed to the presence or absence of maternally-derived anti-PVM antibodies. However, there is a substantial literature on the extra-pulmonary manifestations of RSV infection [reviewed in [23,24]]. For example, RSV infection in human infants is clearly associated with an increased incidence of cardiac arrhythmias [25]. RSV infection also correlates with an increased incidence of central apnea, without any specific association to the ensuing inflammatory response [26]; the link between RSV and apnea has been noted with respect to the link between virus infection and sudden infant death syndrome [27]. Furthermore, a recent study of post-mortem lung tissue by Welliver and colleagues [28] points to a potential role for epithelial cell apoptosis; Bem and colleagues [29] have noted that there are elevated levels of biologically-active soluble TNF-related apoptosis-inducing ligand (sTRAIL) in BAL fluids from infants mechanically-ventilated due to severe RSV infection.

Any one or all of these factors combined may promote weight loss, systemic symptoms, and even death in the absence of inflammatory pathology in the lung.

## Conclusions

PVM infection presents in an atypical fashion in neonatal mice. Although virus replication proceeds indistinguishably when compared to older mice, chemokine production is minimal in lung tissue of neonatal mice and recruitment of proinflammatory leukocytes is likewise diminished. Interestingly, despite diminished inflammatory responses, neonatal mice exhibit failure to thrive, with a markedly diminished weight gain for age similar to virus-infected newborn humans. A systematic study of early responses to PVM infection in newborn mice will provide further insights into the ontogeny of the innate immune response and ultimately a better understanding of the mechanisms involved in neonatal RSV infection.

## Methods

### Mice

Specific pathogen-free C57Black/6 breeding pairs were purchased from Taconic Laboratories (Rockville, MD). These mice remained seronegative for pneumonia virus of mice (PVM) antigens while in use as breeders. For experiments in which newborn mice were inoculated with PVM prior to weaning (hereafter described as neonatal mice), the adult breeder pair was retired, and not used to generate offspring for additional experiments. Each experiment included at least four mice per datapoint, and all experiments were performed three or four times. Clinical symptoms and weights were recorded daily.

### Virus

Virus stocks of mouse-passaged PVM strain J3666 stored in liquid nitrogen were diluted 1:1000 in PBS to a final concentration of 200 plaque forming units (pfu [30])/10 μL. Mice were inoculated intra-nasally with 10 μL PVM in PBS or 10 μL of PBS alone and were evaluated immediately following inoculation (day 0) or on days 4 or 7 thereafter. Virus recovery from lung tissue was determined by a quantitative RT-PCR assay targeting the PVM small hydrophobic (SH) gene as previously described [31], and expressed as copies PVM SH gene per copies cellular GAPDH (PVM_SH_/10^6 ^GAPDH).

### Preparation of single cell suspensions from lung tissue and flow cytometry

Mice were sacrificed by cervical dislocation under isoflurane anesthesia. Lungs were perfused *in situ *by injecting the right ventricle with 0.01 M EDTA in PBS to flush out circulating blood cells. Perfused lungs were removed by dissection and placed into 2 ml RPMI 1640 with 5% fetal bovine serum (FBS). The lungs were teased and cut into pieces and then digested with 3 mL RPMI with 5% FBS, 20 μg/mL DNAse I and 2 mg/mL collagenase D (digestion media). The lungs were then washed in additional digestion media and incubated at 37°C with rocking for 90 minutes. Halfway through the digestion time, 2 mL fresh digestion medium was added. After an additional 90 minutes, digests were placed on ice, and EDTA was added to a final concentration of 10 mM. After 5 minutes, the preparations were strained through a 60 micron cell strainer over a conical tube. The sample was collected via centrifugation, and the remaining red blood cells lysed with 5 mL ammonium chloride sodium bicarbonate (ACK) buffer. Following a 5 minute lysis, the cells were washed twice in Wuerzburg buffer (0.3% BSA in PBS containing 0.005 M EDTA and DNase I), then twice in Hanks balanced salt solution. Isolated lung cells were counted and stained for flow cytometry using the following antibodies and dilutions (all from Becton Dickinson (BD) Biosciences Rutherford, NJ) CD11c-APC at 1:100, CD19-APC at 1:200, CD11b-APCCy7 at 1:400, Gr1-APCCy7 at 1:200, CD4-APCCy7 at 1:100, CD80-PE at 1:100, Mac3-PE at 1:100, CD11b-PE at 1:200, CD8-PE at 1:50, NK1.1-PE at 1:50, CD45-PECy7 at 1:1600, MHCII-FITC at 1:100, CD103-FITC at 1:100, B220-FITC at 1:100, and CD3e-FITC at 1:50, all after blocking with anti-FcγIII/II receptor antibody. Data were collected on an LSRII flow cytometer (BD Biosciences); live cells were analyzed by gating on forward-side scatter. Data were acquired using FACSDIVA software (BD Biosciences) and populations analyzed with FlowJo version 8.7.3 (Tree Star, Inc. Ashland, OR).

### Detection of transcripts encoding proinflammatory mediators

One μg of total RNA extracted from lungs of PVM- or diluent control- inoculated mice (day 7, n = 4 mice per point) was used to perform RT^2 ^*Profiler*(tm) PCR Arrays, using the mouse inflammatory cytokines and receptors platform (PCR Superarray, SA Biosciences Corporation, Frederick MD) as per manufacturer's instructions. First strand cDNA was used for real-time PCR detection of transcripts encoding cytokines, chemokines and related inflammatory mediators and 5 housekeeping genes; controls for genomic DNA contamination, reverse transcription, and PCR amplification were included. All threshold values equal to or greater than 35 were considered as negative. The average value of all housekeeping genes was calculated to establish baseline expression, and ΔC_t _was determined by subtracting the mean C_t _for the housekeeping genes from the C_t _for each transcript of interest. The ΔΔC_t _was calculated for each gene across two groups [ΔC_t _(experimental group) - ΔCt (control group)]. Fold change was then determined by calculating 2^(-ΔΔCt)^.

### Detection of immunoreactive pro-inflammatory mediators in response to PVM infection

Perfused lungs removed from PVM- and diluent-control inoculated mice were blade-homogenized into 1 mL PBS. Cytokines were detected using commercial ELISA kits (R&D Systems, Minneapolis, MN). Protein concentration in each sample was determined by BCA assay.

### Histopathology

On day 7, lungs of sacrificed mice were inflated trans-tracheally using 250 μL 10% phosphate-buffered formalin. The lungs and heart were removed and fixed overnight in 10% phosphate-buffered formalin at 4°C. Samples were paraffin-embedded, sectioned, and stained with hematoxylin and eosin (Histoserv, Inc., Germantown, MD).

### Statistical analysis

Data were analyzed by ANOVA with post-hoc analysis or Student's t-test as appropriate. Outlier datapoints were assessed by Grubb's test.

## List of Abbreviations

IFNγ: interferon gamma; IL: interleukin; MyD88: myeloid differentiation primary response gene 88; PFU: plaque forming unit; PVM: pneumonia virus of mice; RSV: respiratory syncytial virus; SH: small hydrophobic (protein); TLR: toll-like receptor; TNF: tumor necrosis factor;

## Competing interests

The authors declare that they have no competing interests.

## Authors' Contributions

All authors have read and approved the final version of this manuscript. **CAB **performed the virus inoculations, qPCR for cytokine detection and clinical evaluations on all mice evaluated in this study. **CP **generated the single cell suspensions from lung tissue and performed flow cytometric analysis on recruited leukocytes while at SUNY Syracuse. **CMP **determined virus recovery quantitative by qPCR in all lung tissue samples. **HFR **assisted with experimental design, design of display items, and writing of first and all subsequent drafts of the manuscripts. **JBD **conceived and designed the study, collated data and assembled first draft of the manuscript. All authors read an approved the final draft.

## Authors' Information

Dr. Joseph B. Domachowske is a Professor of Pediatrics, Microbiology, and Immunology at State University of New York Upstate Medical University, Syracuse, New York. Dr. Helene F. Rosenberg is Senior Investigator and Section Chief, Laboratory of Allergic Diseases, National Institute of Allergy and Infectious Diseases, Bethesda, Maryland. Drs. Domachowske and Rosenberg are long-time collaborators with shared interests in inflammation and pathogenesis of respiratory virus infection.
